# Exploring the translational potential of PLGA nanoparticles for intra-articular rapamycin delivery in osteoarthritis therapy

**DOI:** 10.1186/s12951-023-02118-4

**Published:** 2023-10-04

**Authors:** Jian-Chao Ma, Tingting Luo, Binyang Feng, Zicheng Huang, Yiqing Zhang, Hanqing Huang, Xiao Yang, Jing Wen, Xiaochun Bai, Zhong-Kai Cui

**Affiliations:** Department of Cell Biology, School of Basic Medical Sciences, Southern Medical University, Guangzhou, 510515 China

**Keywords:** PLGA nanoparticles, Rapamycin, Intra-articular injection, mTORC1, Osteoarthritis therapy

## Abstract

Osteoarthritis (OA) is a prevalent joint disease that affects all the tissues within the joint and currently lacks disease-modifying treatments in clinical practice. Despite the potential of rapamycin for OA disease alleviation, its clinical application is hindered by the challenge of achieving therapeutic concentrations, which necessitates multiple injections per week. To address this issue, rapamycin was loaded into poly(lactic-co-glycolic acid) nanoparticles (RNPs), which are nontoxic, have a high encapsulation efficiency and exhibit sustained release properties for OA treatment. The RNPs were found to promote chondrogenic differentiation of ATDC5 cells and prevent senescence caused by oxidative stress in primary mouse articular chondrocytes. Moreover, RNPs were capable to alleviate metabolism homeostatic imbalance of primary mouse articular chondrocytes in both monolayer and 3D cultures under inflammatory or oxidative stress. In the mouse destabilization of the medial meniscus (DMM) model, intra-articular injection of RNPs effectively mitigated joint cartilage destruction, osteophyte formation, chondrocytes hypertrophy, synovial inflammation, and pain. Our study demonstrates the feasibility of using RNPs as a potential clinically translational therapy to prevent the progression of post-traumatic OA.

**Graphical abstract:**

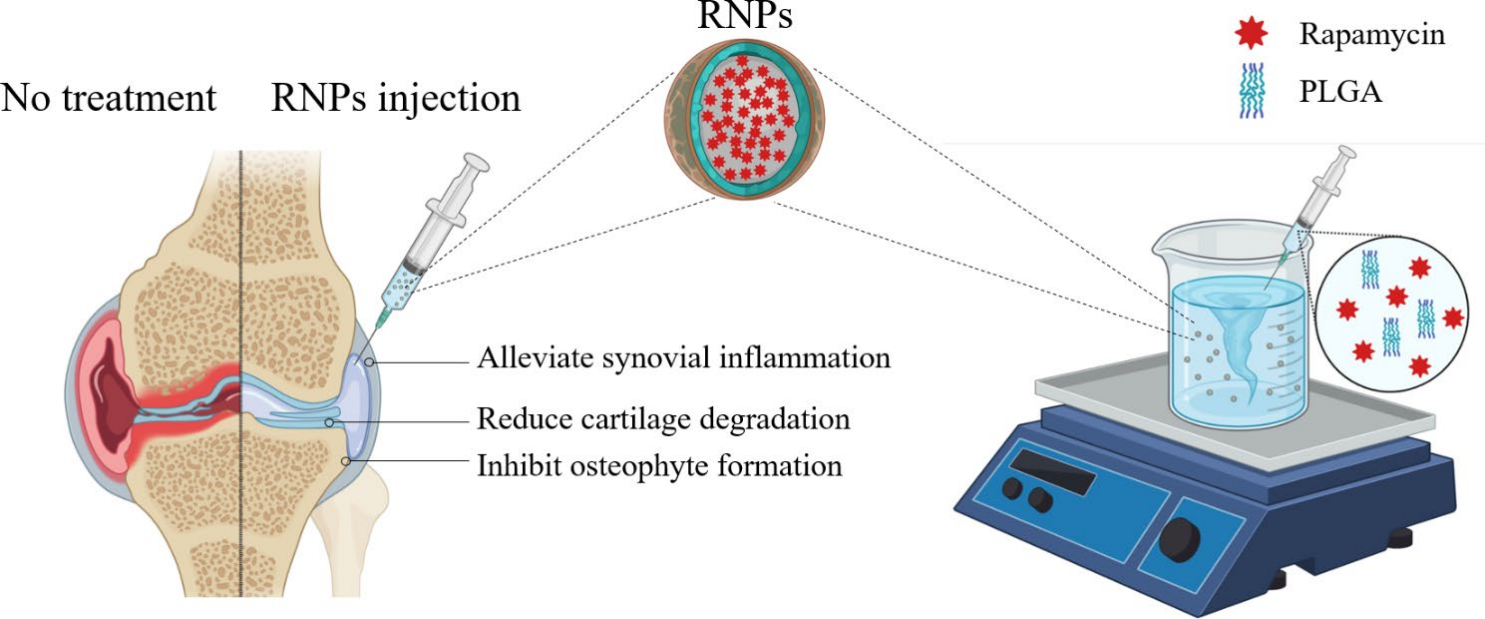

**Supplementary Information:**

The online version contains supplementary material available at 10.1186/s12951-023-02118-4.

## Introduction

Osteoarthritis (OA) is the most common chronic degenerative joint disease, leading to physical disability and posing a significant clinical and financial burden globally. More than 500 million people were affected in 2020 [1, 2]. OA is characterized by the progressive deterioration of articular cartilage, vascular invasion of the articular surface, subchondral bone remodeling, osteophyte formation, synovial inflammation, and pain. Although risk factors, such as genetic predisposition [3], mechanical abnormality [4], age [5] and obesity [6], have been identified, the exact pathogenesis of OA remains unclear. While current clinical treatment strategies, such as non-steroidal anti-inflammatory drugs (NSAIDs) and steroids, can alleviate pain or improve function [7], no known cures or disease-modifying therapies are available yet. Since OA affects all joint tissues [8], it is now considered as a whole joint disease. Consequently, an effective strategy to treat the whole joint is urgently needed instead of targeting the lesions of a certain tissue.

Cartilage degeneration and the progression of OA are strongly influenced by environmental stress, such as inflammation and oxidation [9, 10]. Targeting these stressors may offer novel therapeutic options for modifying the outcomes of OA [11]. One effective target is the mammalian target of rapamycin complex 1 (mTORC1), which can be inhibited by rapamycin, an FDA approved immunosuppressant used to prevent renal transplant rejection for over two decades [12]. In mouse models, rapamycin has been shown to prevent the progression of OA, requiring twice-weekly injections [13]. Furthermore, rapamycin has been verified to alleviate the effects of inflammatory and oxidative stress in many other diseases as well [14]. Intra-articular injection of rapamycin has also been shown to delay the development of OA [15] by acting on chondrocytes degeneration [16], synovial inflammation [17], subchondral osteosclerosis and osteophyte formation [18].

Rapamycin has demonstrated promise as an efficacious drug for treating OA [19]. However, two challenges impede its application in OA treatment. Firstly, the highly hydrophobic nature of free rapamycin results in low solubility in water, only 2.6 µg/mL at 25 °C. Additionally, its macrolide core opens under aqueous conditions, rendering it unstable for intra-articular use [20]. Secondly, effective lymphatic clearance in joints necessitates high-dose administration and frequent injections, which can lead to pain, infection, inflammation, and non-compliance with treatment by patients [21]. Consequently, there is an urgent need for sustained drug release systems that can load rapamycin uniformly dispersed in aqueous solution and substantially prolong its residence time in the joint to fully leverage the potential of intra-articular drug administration for OA treatment. Current research is focused on developing biomaterial-based nanoparticles and hydrogels that can remain in the joint longer and release drugs progressively as required [22].

Poly (_D, L_-lactic-co-glycolic acid) (PLGA) nanoparticles have been recognized as one of the most successful drug delivery systems (DDS) both in laboratories and clinics, mainly due to their predictable degradation in the body that avoids accumulation in the organism [23]. Their well-defined structures also reduce the risk of toxicity or immunogenicity [24]. Therefore, PLGA offers many advantages, including demonstrated safety, low toxicity, and the flexibility of chemical properties due to the tunable ratio and organization of glycolic and lactic monomers [25]. Zilretta®, a PLGA-encapsulated triamcinolone acetonide formulation, has been widely used in clinics to reduce inflammation in OA patients, demonstrating the feasibility of biomaterial-based delivery for OA [26]. Nevertheless, the efficacy of rapamycin-PLGA nanoparticles (RNPs) for the treatment of OA in vivo has yet to be explored. The use of RNPs could potentially overcome the drawbacks of free rapamycin by enabling sustained drug release, reducing the frequency of injections, and minimizing systemic exposure. Moreover, the use of RNPs may result in better distribution of the drug in the joint and reduce the risk of side effects associated with free drug injections.

An ideal intra-articular injection formulation should have the ability to remain in the joint cavity for an extended period and disperse easily, forming a stable dispersion system in the injection solution [27]. For prolonged residence in the joint, particle size is a decisive parameter for treatment efficacy as it directly influences drug retention in the joint. Nanoparticles with sizes larger than 250 nm can prevent rapid clearance from the joint cavity across blood capillaries [28, 29]. Conversely, large particles such as microspheres (> 1000 nm) are challenging to disperse in aqueous solutions, require dispersion by ultrasound or rigorously stirring, and are prone to precipitation, making it difficult to form a stable dispersion system. Additionally, PLGA microspheres formulations require large-diameter gauge needles for injection, which is not conducive to intra-articular injection applications [30, 31].

Herein, we demonstrate a robust drug carrier platform based on PLGA that effectively encapsulates rapamycin and can be tuned to release the drug at varying rates for more than one week. The size of the resulting RNPs ranges from 250 to 450 nm, with an encapsulation efficiency up to 85.2%. The encapsulated rapamycin remained active and can promote chondrogenic differentiation of ATDC5 cells. In addition, the RNPs enhance anabolism and reduce catabolism of primary articular chondrocytes under different stress conditions, both in monolayer and 3D cultures. Moreover, the RNPs formulation effectively ameliorates trauma-induced OA in mice. Taken together, this approach presents a promising, clinically translational therapy to prevent the progression of OA.

## Results

### RNPs fabrication and characterization

**Fig. 1:**
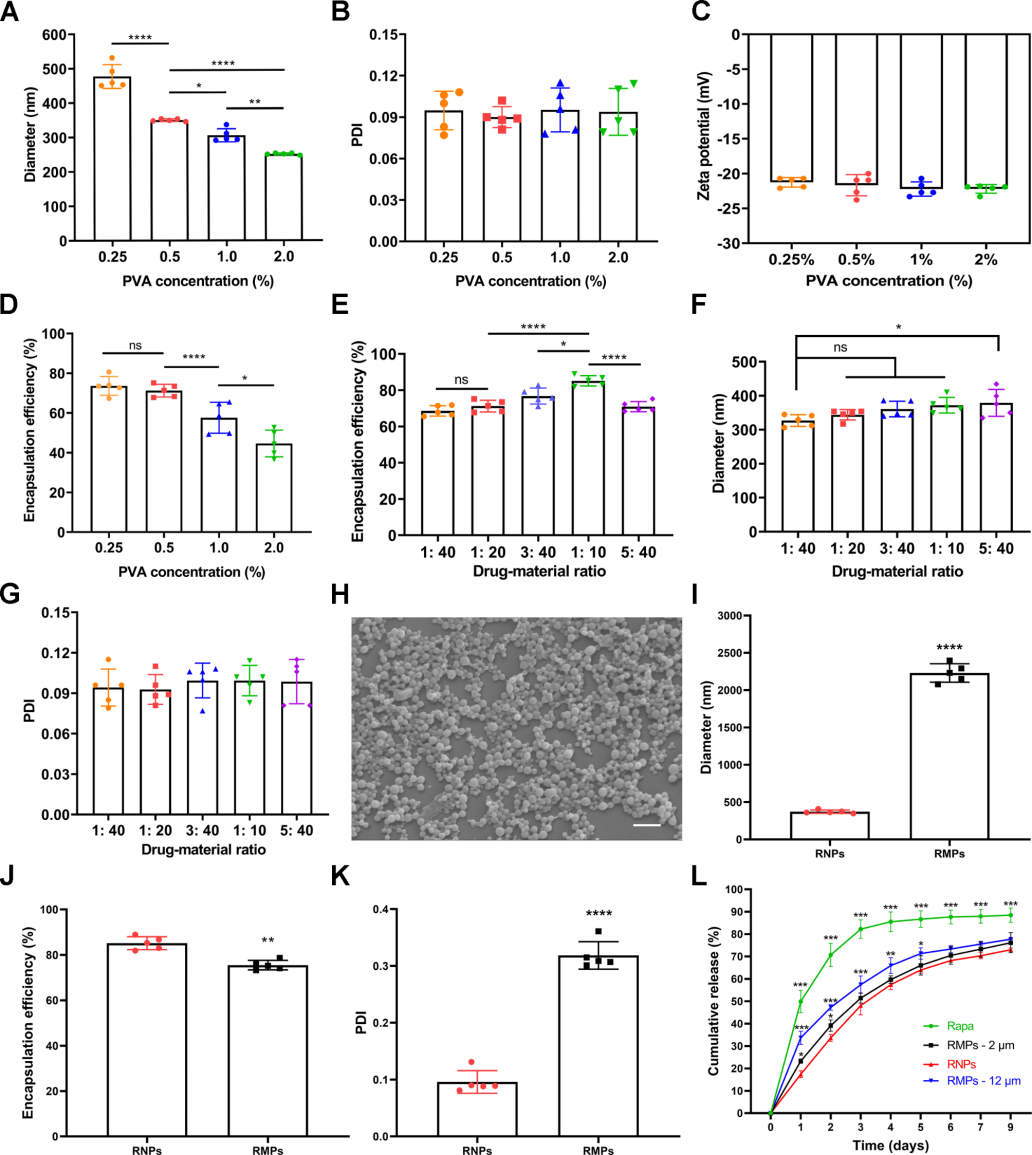
Preparation and characterization of RNPs and RMPs. (**A**) Average diameter of RNPs with different PVA concentrations measured by dynamic light scattering. (**B**) PDI of RNPs with different PVA concentrations. (**C**) Zeta potential of RNPs with different PVA concentrations. (**D**) Encapsulation efficiency of RNPs with different PVA concentrations measured by HPLC. (**E**) Encapsulation efficiency of RNPs with different initial drug-to-material ratios measured by HPLC. (**F**) Average diameter of RNPs with different initial drug-to-material ratios measured by dynamic light scattering. (**G**) PDI of RNPs with different initial drug-to-material ratios. (**H**) Scanning electron micrograph of RNPs, Scale Bar 1 μm. (**I-K**) Average diameter, encapsulation efficiency, PDI of RNPs and RMPs (0.5% PVA concentration, drug-to-material ratio of 1:10). (**L**) Quantification of in vitro release profiles of Rapa, RMPs-12 μm, RMPs-2 μm and RNPs groups over 9 days. n = 3. Statistical analysis was performed using one-way ANOVA with Tukey’s post hoc analysis. Data are presented as means ± SD. ns, no significant. *P < 0.05, **P < 0.01, ***P < 0.001, ****P < 0.0001

RNPs were synthesized using the emulsification and volatilization method, with dynamic light scattering for an average diameter range of 250–470 nm. Polyvinyl alcohol (PVA) was used as a water-soluble surfactant to facilitate particle formation, and its concentration was found to significantly impact particle size, with no nanoparticles forming at concentrations below 0.25%. As the concentration of PVA increased, the size of the nanoparticles decreased (Fig. 1A). The polydispersity index (PDI, ~ 0.100) and zeta potential (< -20 mV) of the nanoparticles remained nearly constant over the investigated PVA concentration range (Fig. 1B, C). Encapsulation efficiency decreased as PVA concentration increased above 0.5%, with no significant difference between 0.5% and 0.25% PVA concentration (Fig. 1D). The maximum encapsulation efficiency of rapamycin (85.2% ± 3.2) was achieved at 0.5% PVA concentration and a drug-to-material ratio of 1:10 (w/w) (Fig. 1E), with lower or higher drug-material ratios leading to decreased efficiency. Loading capacity is approximately 7.8% at the condition of 0.5% PVA concentration and an initial drug-to-material ratio of 1:10 (w/w). The particle size, PDI and zeta potential remained constant, except when the initial drug-to-material ratio exceeded 1:10, resulting in nanoparticle enlargement (Fig. 1F, G&S1A). Spherical nanoparticles with a smooth surface, a uniformly distribution and an average hydrodynamic diameter of approximately 350 nm at 0.5% PVA and 1:10 drug-to-material ratio, which was further confirmed by scanning electron microscopy (Fig. 1H).

Rapamycin-PLGA microspheres (RMPs) ranging from 2 to 12 μm were successfully formulated under appropriate conditions in addition to nanoparticles (Figure S1B). Unfortunately, with the larger size (Fig. 1I&S1C-F), the encapsulation efficiency did not increase concomitantly (Fig. 1J), and loading capacity was only about 7.0%. Significantly higher PDI (> 0.300) was observed compared to that of nanoparticles (Fig. 1K), and ~ 30 mV for zeta potential (Figure S1G). Moreover, the stability of RMPs suspension in physiological saline was inferior to that of RNPs (Figure S2). The drug release profiles of free rapamycin (Rapa), RNPs and RMPs were measured using the dialysis method (Fig. 1L). An abrupt release of ~ 50% rapamycin was observed for the Rapa group within the first day, while only 33%, 23% and 17% were released from the RMPs-12 μm, RMPs-2 μm and RNPs groups, respectively. Within 3 days, 82% rapamycin was released from the Rapa group, while 75%, 73% and 70% were released from the RMPs-12 μm, RMPs-2 μm and RNPs, respectively, within one week. Both microspheres and nanoparticles acted as slow-release rapamycin platforms under in vitro conditions, with nanoparticles significantly reducing the initial burst, having a higher encapsulation efficiency and loading capacity of rapamycin compared to microspheres.

### NPs achieve higher cellular uptake efficiency and biocompatibility

**Fig. 2:**
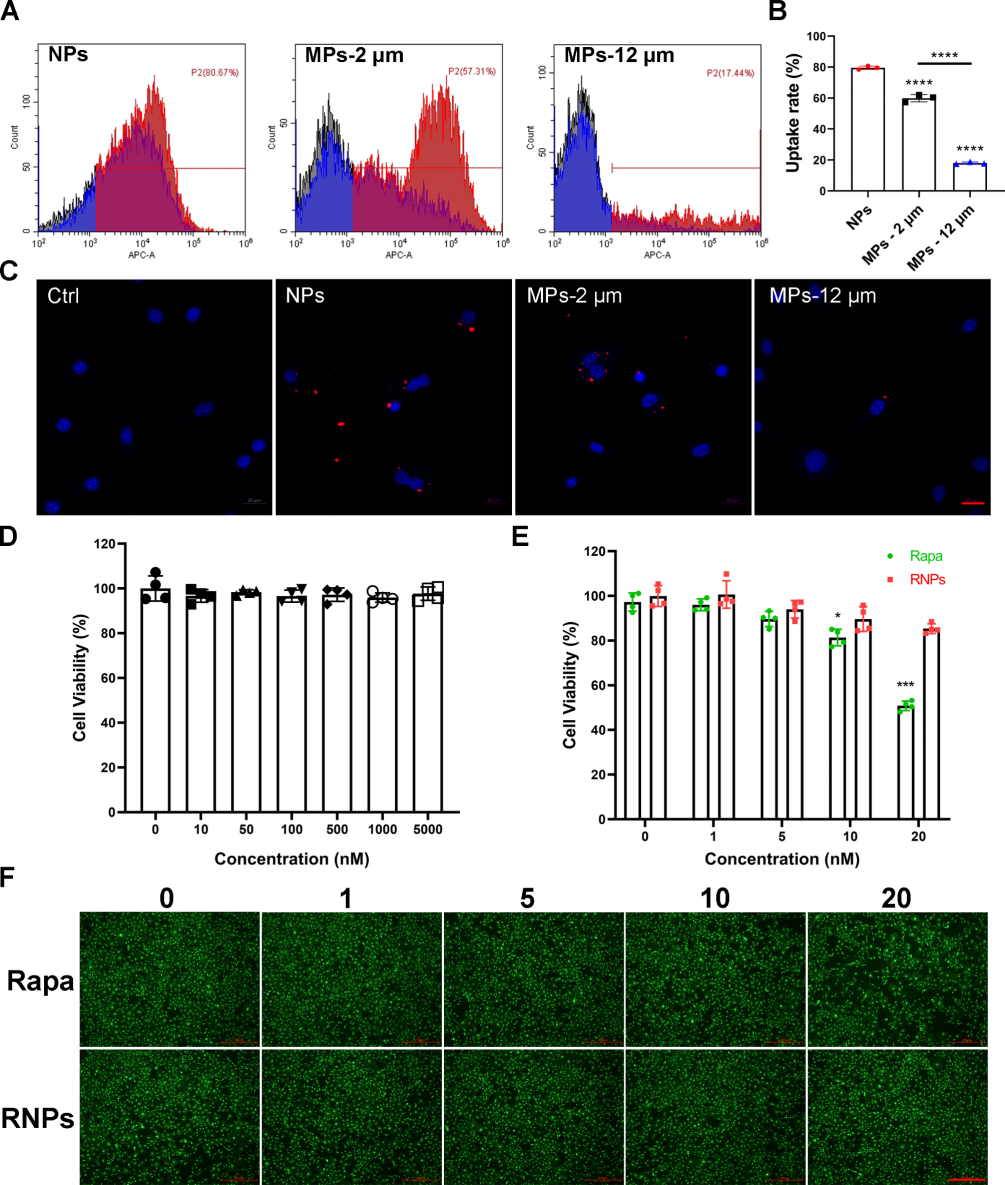
In vitro cellular uptake and biocompatibility. (**A, B**) Cellular uptake of RNPs and RMPs by primary articular chondrocytes measured by flow cytometry (n = 3). (**C**) Cellular uptake of RNPs and RMPs visualized by CLSM, Scale Bar 20 μm. (**D**) Cell viability of primary articular chondrocytes cultured with different concentrations of NPs measured by CCK8 assay (n = 4). (**E**) Cell viability of primary articular chondrocytes cultured with different concentrations of free rapamycin and RNPs measured by CCK8 assay at 48 h (n = 4). (**F**) Fluorescence images of Live/Dead staining on primary articular chondrocytes cultured with different concentrations of free rapamycin and RNPs, Scale Bar 500 μm. Statistical analysis was performed using one-way ANOVA with Tukey’s *post hoc* analysis. Data are presented as means ± SD. *P < 0.05, **P < 0.01, ***P < 0.001, ****P < 0.0001

The effectiveness of rapamycin in inhibiting the mTORC1 signaling pathway depends on its ability to reach the cytosol. Therefore, the uptake efficiency of the NPs and MPs was evaluated using flow cytometry and confocal laser scanning microscope (CLSM) after a 12 h incubation. As depicted in Fig. 2A&B, the cellular uptake rate of DID-labeled NPs exceeded 80%, whereas the average cellular uptake rate of MPs with different sizes was only 57% and 17% respectively. Consistent results were obtained with the mouse leukemic monocyte/macrophage cell line (RAW 264.7) as well (Figure S3). CLSM images further confirmed that primary articular chondrocytes readily internalized DID-labeled NPs (Red), while the uptake rate was significantly lower for MPs (Fig. 2C, Figure S4). The nanoscale size of NPs, which provides a larger surface area, facilitates more efficient interactions with cells and allows for easier passage across cellular barriers compared to the larger microspheres. Therefore, 350 nm nanoparticles were selected for further investigation in OA therapy.

To evaluate the cytotoxicity of NPs, Rapa and RNPs on primary articular chondrocytes, a CCK8 assay and Live/Dead staining were carried out. Figure 2D indicates that even at a high concentration of 5 µM, NPs did not exhibit any detectable cytotoxicity against primary articular chondrocytes. At a concentration of 20 nM, the cell viability value for Rapa and RNPs was 50.8% and 85.3%, respectively (Fig. 2E), with Live/Dead staining showing fewer cells in the Rapa group. Live/Dead staining did not reveal the presence of dead cells (red) probably due to rapamycin-mediated inhibition of cell metabolism (Fig. 2F). The dose-dependent cytotoxicity of both Rapa and RNPs to primary articular chondrocytes was observed, with RNPs exhibiting significantly lower cytotoxicity than Rapa at the same concentration. These results suggest that RNPs are a promising candidate for cartilage-targeted rapamycin delivery applications.

### RNPs promote chondrogenic differentiation

**Fig. 3:**
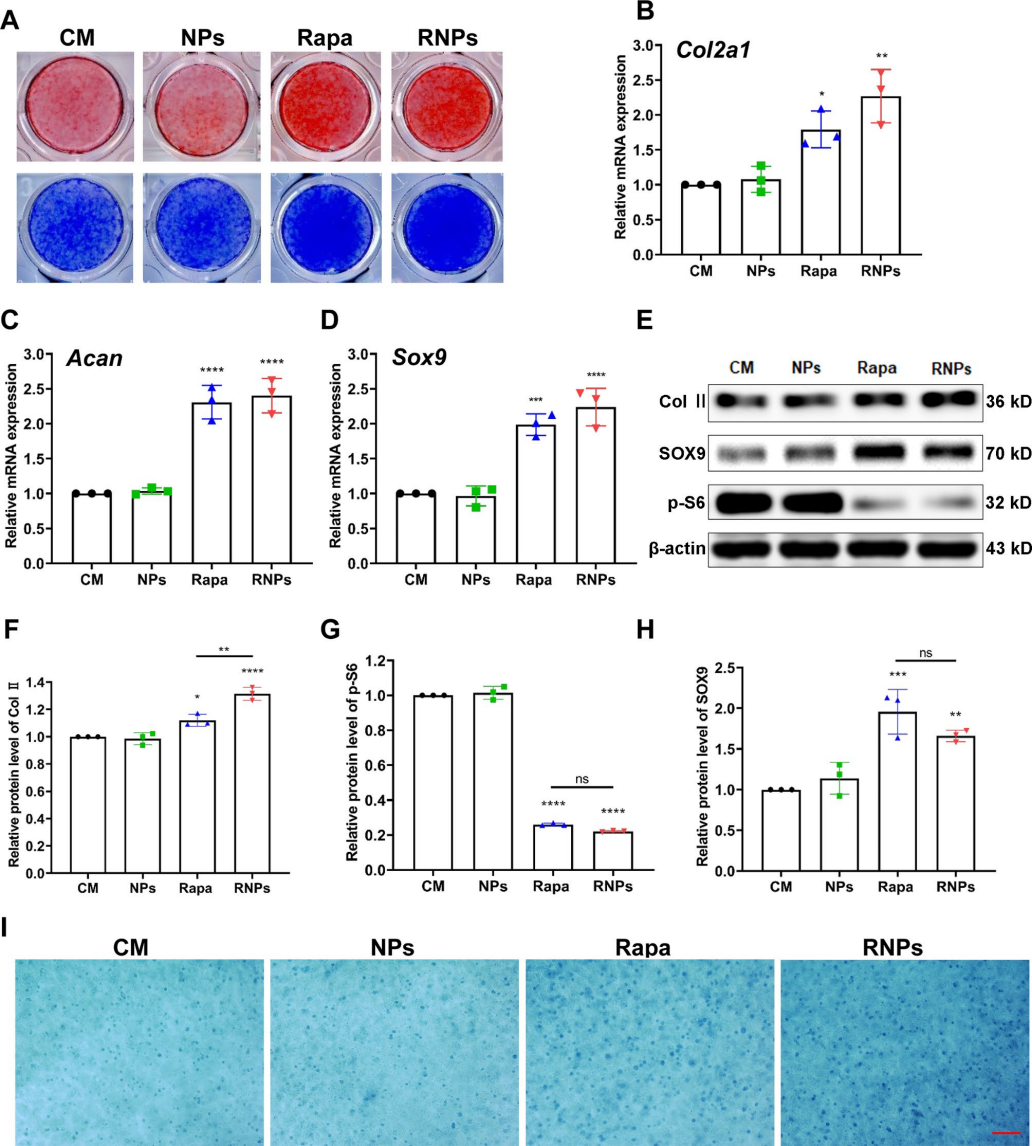
RNPs promote chondrogenic differentiation of ATDC5 cells. (**A**) Safranin O and Toluidine blue staining of ATDC5 cells cultured in chondrogenic medium for 7 days. (**B-D**) The expression of chondrogenic genes *Col2a1*, *Acan* and *Sox9* in ATDC5 cells after 96 h of chondrogenic differentiation. (**E-H**) The expression of relative proteins (Col II, SOX9 and p-S6) of ATDC5 cells cultured in chondrogenic medium for 7 days. (**I**) Alcian blue staining of ATDC5 cells encapsulated in hydrogels for chondrogenic differentiation and cultured in chondrogenic medium for 14 days, Scale Bar 200 μm. n = 3 per group. Statistical analysis was performed using one-way ANOVA with Tukey’s *post hoc* analysis. Data are presented as means ± SD. *P < 0.05, **P < 0.01, ***P < 0.001, ****P < 0.0001

To further explore the effect of RNPs on chondrogenesis, ATDC5 cells were utilized to evaluate their impact on chondrogenic differentiation. ATDC5 cells were treated with varying concentrations of rapamycin and incubated for 7 days. Toluidine blue staining exhibited an intensified color with increasing rapamycin concentration up to 20 nM, followed by a decrease at 30 nM. The most potent chondrogenic differentiation enhancement of rapamycin was considered at 20 nM (Figure S5). Safranin O and Toluidine blue staining revealed that NPs did not affect the chondrogenic differentiation process of ATDC5 cells. Both rapamycin and RNPs substantially promoted chondrogenic differentiation at the concentration of 20 nM (Fig. 3A), which was consistent with the significant upregulation of chondrogenic genes *Col2a1*, *Acan* and *Sox9* (Fig. 3B-D). Further examination of the expression of chondrogenic differentiation-related proteins Col II and SOX9 resulted in consistency, and a reduction in p-S6 expression indicated effective inhibition of the mTORC1 signaling (Fig. 3E-H). To simulate chondrogenic differentiation in vivo, ATDC5 cells and rapamycin or RNPs were encapsulated in the methacrylate glycol chitosan (MeGC) hydrogel developed in our previous work [32] for chondrogenic differentiation. After a 14-day incubation period, Alcian blue staining demonstrated that both rapamycin and RNPs promoted chondrogenic differentiation. Moreover, the chondrogenic differentiation efficacy of RNPs was superior to that of free rapamycin (Fig. 3I).

### RNPs alleviate IL-1β-induced catabolic homeostatic imbalance and enhance anabolic response in chondrocytes

**Fig. 4:**
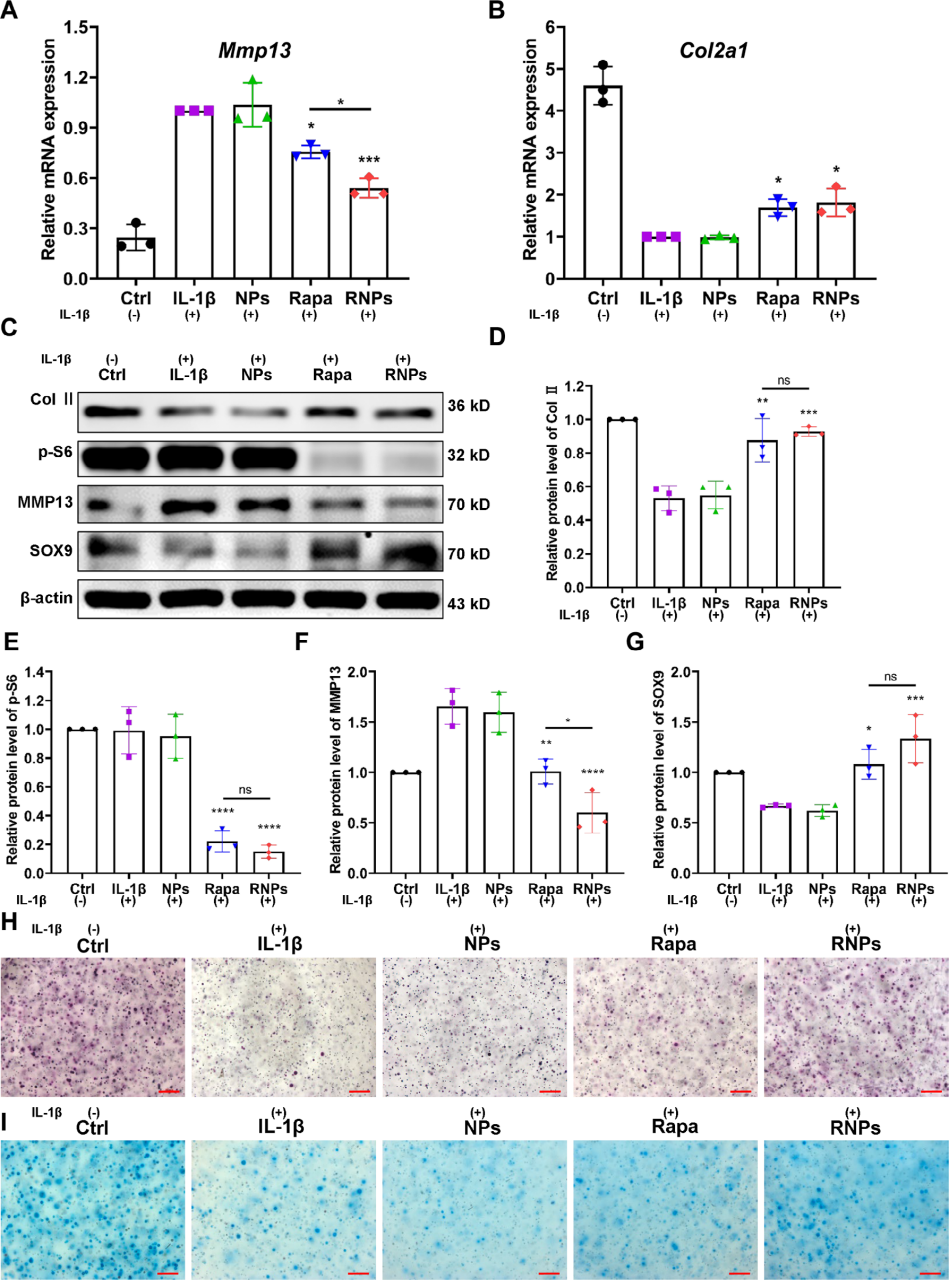
RNPs alleviate IL-1β-induced metabolic homeostatic imbalance in chondrocytes. The expression levels of the catabolic marker *Mmp13* (**A**) and the cartilage-specific marker *Col2a1* (**B**) in primary articular chondrocytes, which were induced with IL-1β, except the Ctrl group, the other groups were treated with IL-1β for 24 h. (**C-G**) The expression of proteins (Col II, SOX9, MMP13 and p-S6) in primary articular chondrocytes, which were induced with IL-1β for 24 h. Toluidine blue (**H**) and Alcian blue (**I**) staining of primary articular chondrocytes, encapsulated in hydrogels and induced with IL-1β for 24 h, Scale Bar 200 μm. n = 3. Statistical analysis was performed using one-way ANOVA with Tukey’s *post hoc* analysis. Data are presented as means ± SD. *P < 0.05, **P < 0.01, ***P < 0.001, ****P < 0.0001

The impact of RNPs on IL-1β-induced metabolic imbalance in chondrocytes was assessed by examining the gene expression levels of the catabolic marker (*Mmp13*) and the cartilage-specific marker *Col2a1* after a 48-h incubation period. The expression of *Mmp13* in the IL-1β group was significantly upregulated, with a remarkable ~ 4-fold increase compared to the control group (Fig. 4A). The administration of NPs alone did not result in any changes. However, treatment with either rapamycin or RNPs led to a decline in the *Mmp13* expression, with RNPs exhibiting the most pronounced reduction of about 50%. Simultaneously, RNPs upregulated the expression of the cartilage-specific gene of *Col2a1* by 1.8-fold compared to the IL-1β group (Fig. 4B). Moreover, after 7 days of culture, a significant upregulation of the expression of anabolic-related proteins Col II and SOX9, along with a downregulation of catabolic-related protein expression (MMP13) was observed (Fig. 4C-G). Consistent results were obtained with human chondrosarcoma cells (SW1353) as well (Figure S6). Then primary articular chondrocytes were encapsulated with either rapamycin or RNPs in hydrogels, induced with IL-1β for 24 h, and then incubated for 7 days. Toluidine blue and Alcian blue staining demonstrated that both rapamycin and RNPs alleviated IL-1β-induced damage to primary articular chondrocytes (Fig. 4H, I). Notably, the rescue effect of RNPs was superior to that of free rapamycin (Figure S7).

### RNPs prevent H_2_O_2_-induced metabolic homeostatic imbalance and senescence in chondrocytes

**Fig. 5:**
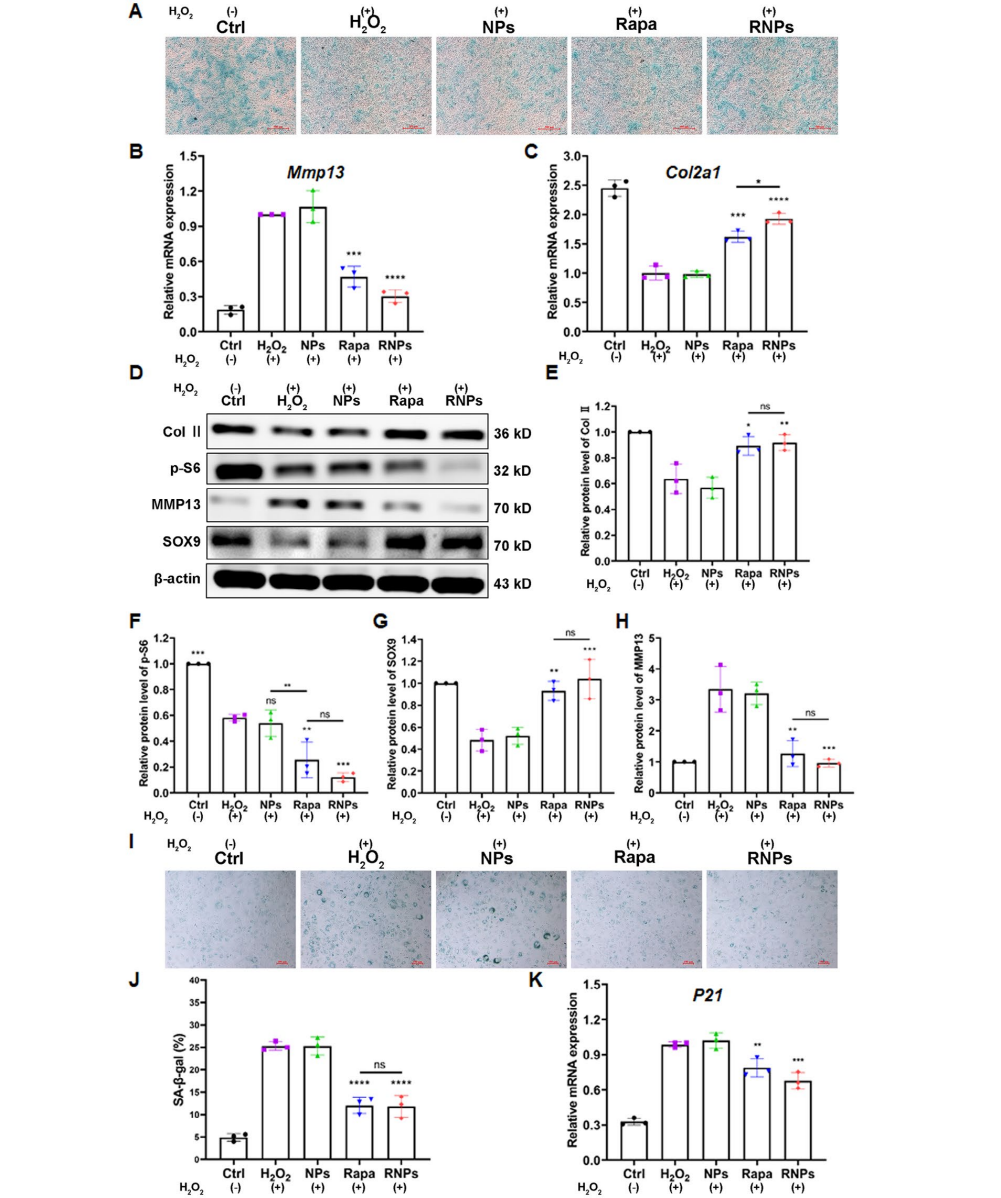
RNPs prevent H_2_O_2_-induced metabolic homeostatic imbalance and prevent senescence in chondrocytes. (**A**) Alcian blue staining of primary articular chondrocytes, treated with 200 µM H_2_O_2_ for 24 h and maintained in 50 µM H_2_O_2_ for an additional week in the absence or presence of NPs, Rapamycin and RNPs, Scale Bar 500 μm. Except the Ctrl group, the other groups were treated with H_2_O_2_. (**B, C**) The expression of the catabolic marker *Mmp13* (n = 3) and the cartilage-specific marker *Col2a1* (n = 3) in primary articular chondrocytes induced with 200 µM H_2_O_2_. (**D-H**) The expression of relative proteins (Col II, SOX9, MMP13 and p-S6) in primary articular chondrocytes induced with 200 µM H_2_O_2_. (**I**) SA-β-gal staining of primary articular chondrocytes after H_2_O_2_-induction, Scale Bar 100 μm. (**J**) Quantification of SA-β-gal positive primary articular chondrocytes (n = 3). (**K**) The expression of the senescence marker *P21* in primary articular chondrocytes, induced with 200 µM H_2_O_2_ (n = 3). Statistical analysis was performed using one-way ANOVA with Tukey’s *post hoc* analysis. Data are presented as means ± SD. *P < 0.05, **P < 0.01, ***P < 0.001, ****P < 0.0001

Primary articular chondrocytes were exposed to 200 µM H_2_O_2_ for 24 h and then maintained in 50 µM H_2_O_2_ for an additional week in the absence or presence of NPs, rapamycin and RNPs. Alcian blue staining unraveled that the presence of NPs did not affect the metabolic state of primary articular chondrocytes. Under oxidative stress conditions, both rapamycin and RNPs were found to promote anabolism and inhibit catabolism, with RNPs showing a more pronounced effect on the anabolic-catabolic homeostasis of primary articular chondrocytes (Fig. 5A). The effect of RNPs on H_2_O_2_-induced chondrocytes was quantified by analyzing the gene expression levels of *Mmp13* and *Col2a1* after 96 h of culture. The expression of *Mmp13* in the H_2_O_2_ and NPs groups was found to be the highest among all the groups, showing a remarkable increase of ~ 5.7-fold (for *Mmp13*) compared to the control group. Treatment with rapamycin or RNPs resulted in a decline of the *Mmp13* level (Fig. 5B). Particularly, RNPs reduced the H_2_O_2_-stimulated upregulation most prominently, with a decline of ~ 70%. Simultaneously, RNPs upregulated the cartilage-specific gene of *Col2a1* by 1.9-fold compared with the H_2_O_2_ and NPs groups (Fig. 5C). Furthermore, a significant upregulation of the expression of anabolic-related proteins Col II and SOX9, along with a downregulation of the expression of catabolic-related protein MMP13 was detected after treatment with rapamycin or RNPs (Fig. 5D-H). Oxidative stress conditions can promote cellular senescence, and senescence-associated β-galactosidase (SA-β-gal) staining showed a significant increase in primary articular chondrocytes after H_2_O_2_-induced senescence. However, after treatment with rapamycin or RNPs, the level of SA-β-gal was significantly reduced, indicating that these treatments can prevent the senescence of H_2_O_2_-induced primary articular chondrocytes (Fig. 5I, J). The expression of the aging-related gene *P21* further confirmed that RNPs can significantly alleviate the senescence of H_2_O_2_-induced primary articular chondrocytes (Fig. 5K).

### RNPs exhibit prolonged retention in joints

**Fig. 6:**
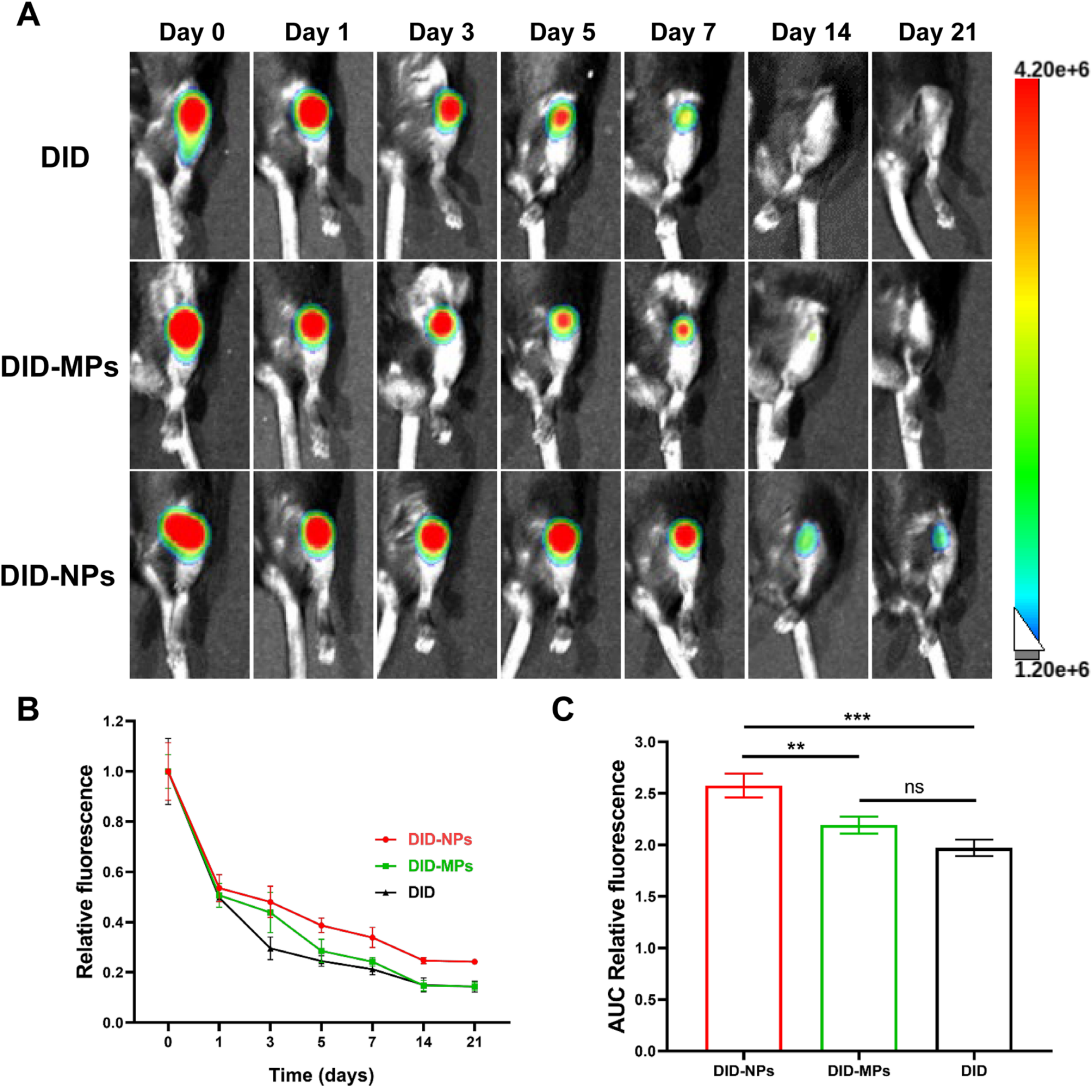
Joint retention of RNPs. (**A**) Representative fluorescence images of knee joints from OA mice over 21 days after intra-articular injection of free DID (DID), DID-labeled MPs (DID-MPs) and DID-labeled NPs (DID-NPs), respectively. (**B**) Quantitative analysis of the time course of relative fluorescence intensity within knee joints after intra-articular injection of free DID (DID), DID-labeled MPs (DID-MPs) and DID-labeled NPs (DID-NPs), respectively. (**C**) Quantitative analysis of the area under the curve (AUC) based on the relative fluorescence intensity profile in (B). n = 5 per group. Statistical analysis was performed using one-way ANOVA with Tukey’s *post hoc* analysis. Data are presented as means ± SD. *ns*, non-significant. *P < 0.05, **P < 0.01, ***P < 0.001, ****P < 0.0001

PLGA nanoparticles labeled with DID were directly injected into the knee joint to investigate their retention under OA conditions. A well-acknowledged post-traumatic OA model of destabilization of the medial meniscus (DMM) was employed. DMM was performed on the joints 30 days before injection to simulate post-traumatic OA. The retention of PLGA nanoparticles was assessed through fluorescence imaging with an in vivo imaging system at day 0, 1, 3, 5, 7, 14 and 21, respectively (Fig. 6A). Results showed that the joints that received PLGA nanoparticles displayed significantly intensified fluorescence signals compared to those injected with DID-MPs or DID over the investigated period, indicating prolonged retention of PLGA nanoparticles (Fig. 6B). Quantitative analysis based on the area under the curve (AUC) of relative fluorescence intensity profiles revealed that NPs significantly enhanced the joint retention ability of rapamycin (Fig. 6C). In addition, the biodistribution analysis of PLGA nanoparticles showed that fluorescence signals were solely detected in the joint 24 h after injection (Figure S8), suggesting that the nanoparticles remain localized in the joint space.

### RNPs manifest therapeutic potential in alleviating knee osteoarthritis in vivo

**Fig. 7:**
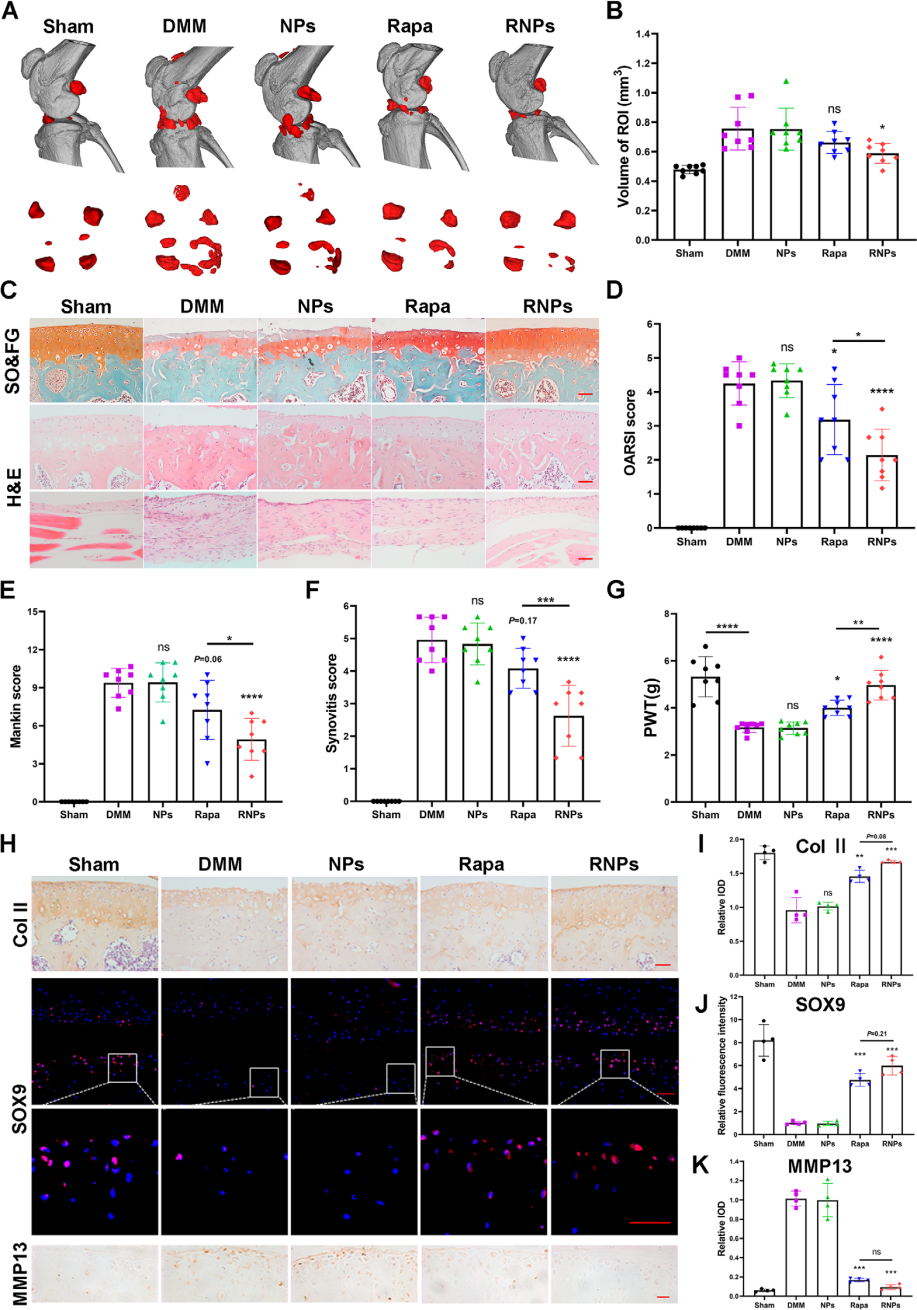
RNPs alleviate knee osteoarthritis in vivo. (**A**) Micro-CT imaging of the morphological structure of the knee in mice at week 8 after surgery. Scale bar = 10 mm. (**B**) Osteophyte maturity (ROI zone) in the different groups of mice at week 8 after surgery, n = 8 mice per group. (**C**) Safranin-O/fast green staining of the mouse knee joint cartilage (hypertrophic chondrocytes were marked with black arrows), hematoxylin and eosin staining of cartilage and synovium at week 8 after surgery, Scale Bar 50 μm. (**D, E**) OA severity of knee joints at week 8 after surgery evaluated by OARSI score (**D**) and Mankin score (E), n = 8 mice per group. (**F**) Synovitis score, n = 8 mice per group. (**G**) Von Frey assay was performed at week 8 after surgery, n = 8 mice per group. (**H**) Immunohistochemical staining to detect the expression of cartilage specific indicators Col II (scale bar 50 μm) and the catabolic marker MMP13 (scale bar 20 μm) in mouse knee cartilage after sham or DMM surgery at week 8. Immunofluorescence staining to detect the expression of the cartilage specific indicator SOX9 (scale bar 20 μm). (**I-K**) Quantitative analysis of the staining images in (**H**), n = 3. Statistical analysis was performed using one-way ANOVA with Tukey’s *post hoc* analysis. Data are presented as means ± SD. *ns*, non-significant. *P < 0.05, **P < 0.01, ***P < 0.001, ****P < 0.0001

To evaluate the therapeutic potential of RNPs, we established DMM-induced OA models in mice, as evidenced by a reduction in the area of Safranin O staining, an increase in the number of hypertrophic chondrocytes and an enhancement of synovial inflammation. The in vitro release profile of rapamycin from RNPs demonstrated a sustained release with 70% of the drug liberated by day 7, which informed our selection of weekly intra-articular injections. To assess the prophylactic potential, NPs, rapamycin (Rapa) and RNPs were administered *via* intra-articular injection, starting one week after DMM surgery. Intra-articular injection of Rapa and RNPs did not affect the main internal organs of mice (Figure S9). RNPs administration significantly inhibited DMM-induced osteophyte formation (Fig. 7A, B). The cartilage in the DMM and NPs groups exhibited surface irregularities and degeneration, including erosion and surface fibrillation. While in the rapamycin treated group, the cartilage surface appeared flatter than in the DMM groups. However, compared to the sham group, significant cartilage erosion, an increase in hypertrophic chondrocytes and synovial hyperplasia were still observed in the Rapa group (Fig. 7C). In contrast, administration of RNPs significantly reduced cartilage surface erosion, prevented the increase in hypertrophic chondrocytes, and alleviated synovial inflammation compared to the sham group. Both rapamycin and RNPs reduced the severity of osteoarthritis, as indicated by the OARSI and Mankin scores (Fig. 7D, E), with the sustained release function of RNPs achieving a more effective osteoarthritis treatment effect. RNPs reduced the severity of synovial inflammation and pain (Fig. 7F, G) as indicated by the synovitis score. Immunohistochemical and immunofluorescence staining for cartilage-specific indicators, Col II and SOX9, respectively, showed that treatment with rapamycin and RNPs rescued the decrease in Col II and SOX9 expression caused by DMM surgery. Consistently, MMP13 expression decreased in the rapamycin and RNPs treatment groups (Fig. 7H-K). Moreover, the treatment effect of RNPs was more efficacious than that of free rapamycin. Overall, our findings indicate that RNPs hold promising therapeutic potential in effectively alleviating knee osteoarthritis in vivo.

## Discussion

Osteoarthritis (OA) is a whole joint degenerative disease that involves the breakdown of articular cartilage, subchondral bone sclerosis, and osteophyte formation. Current treatments in clinics for OA primarily focus on symptom relief, rather than disease modification. Although several small molecule drugs and biological macromolecules have shown promise in modifying OA pathology in animal models, difficulties in drug delivery have hindered their translation into clinical practice [8, 15, 33–35]. Specially, maintaining effective concentrations and preventing rapid clearance of the drug from the joint cavity have been proven challenging. Encapsulation of drugs in slow-degrading polymer matrices has been proposed as one of the strategies to reduce the frequency of administration. In this study, we have developed a robust drug carrier platform using PLGA nanoparticles to encapsulate rapamycin with high encapsulation efficiency (> 85%) and achieved sustained liberation of rapamycin in the joint for OA treatment. Based on physicochemical characterizations, we found that a once-per-week injection of RNPs was sufficient to achieve a satisfactory outcome. Similar frequencies have been used in clinical trials, such as Hyalgan® (3 cycles of weekly IA injections for 5 weeks, NCT00669032) and Enbrel® (weekly IA injections for 5 weeks, NCT02722772). Consequently, we believe that our injection frequency of RNPs is appropriate for clinical applications.

Utilizing a sustained-release system based on polymers may be the key to the clinical translation of OA treatment drugs. However, previous drug delivery studies of OA treatments have selected a variety of mixed materials and/or multiple drugs to achieve functional drug delivery for multi-target treatment [36, 37]. This can be challenging to apply clinically due to their cumbersome preparation processes and complex composition. PLGA is a biomaterial with well-established safety profiles and degradability that was approved for clinical usage [23]. Additionally, rapamycin has been approved in the market for more than 20 years [12]. Therefore, the application of RNPs is a promising approach that can be rapidly translated to clinics.

We have successfully prepared PLGA particles with sizes ranging from 250 nm to 12 μm. Nanoparticles offer several advantages over microspheres, including greater stability in aqueous environments, higher cellular uptake efficiency, and easier dispersion and injection [27, 31]. However, particles smaller than 250 nm may be rapidly cleared from the joint cavity through blood capillaries [28, 29], while particles larger than 500 nm may cause injection site irritation [38]. After optimization, we determined that 350 nm RNPs demonstrated the optimal size and potency for OA treatment in our study. In our animal experiments, we selected nanoparticles fabricated from 50 kDa molecular weight PLGA which exhibited a prolonged residence time of at least 21 days in the knee joints of mice. It is conceivable that using PLGA particles composed of a higher molecular weight polymer, could further extend the residence time in the joints and reduce the frequency of administration [39]. By modifying the molecular weight of the PLGA, one can achieve sustained drug release and maintain therapeutic drug concentrations in the joint. Moreover, this platform can be potentially applied for delivering other promising drugs for OA treatment.

Despite lacking intrinsic reparative ability, articular cartilage contains a population of stem cells or progenitor cells that resemble those found in other adult tissues, and are believed to be play a key role in maintaining tissue homeostasis [40]. Various studies have demonstrated the involvement of cartilage stem/progenitor cells in the development of osteoarthritis, and their potential as a new direction for treatment [41–43]. Our research confirmed that RNPs can effectively promote chondrogenic differentiation of ATDC5 cells, suggesting that they may inhibit the progression of osteoarthritis by promoting the differentiation of cartilage stem/progenitor cells. OA occurs due to an imbalance between the anabolic and catabolic activity of chondrocytes [19, 44, 45]. In stressful environments such as inflammation or increased levels of reactive oxygen species (ROS), chondrocyte anabolism decreases while catabolism increases, which in turn accelerates osteoarthritis progression. Multifaceted approaches such as ROS clearance and inflammation reduction in stressful environments hold promise as translational therapies [10, 46]. In inflammatory and oxidative stress environments, RNPs can reduce the expression of chondrocyte catabolic marker MMP13, while simultaneously increase the expression of chondrocyte anabolism markers Col II and SOX9. The DMM group exhibited a lower number of cells expressing Col II and SOX9, while a higher number of cells secreted the catabolic marker MMP13, compared to the sham group. In contrast, the RNPs treatment groups showed an upregulation of anabolism and a reduction in the expression of MMP13 (Fig. 7H-K). As OA primarily affects the elderly, chondrocyte senescence is another crucial contributing factor in its development [45]. Senescent chondrocytes can adopt a new secretory phenotype, releasing pro-inflammatory cytokines, vascular growth factors, and catabolic enzymes, which transform the phenotype of articular chondrocytes into hypertrophic cells, impairing the extracellular matrix (ECM) stability and leading to the exacerbation of OA [47]. Several drugs, including melatonin [48], metformin [49] and rapamycin [50], have been demonstrated to treat OA by inhibiting cellular aging. Our research indicates that RNPs can safeguard primary articular chondrocytes from oxidative stress, preventing them from entering senescence and acquiring the pro-inflammatory and catabolic phenotype associated with OA.

In conclusions, we have successfully developed and optimized an efficacious rapamycin-PLGA nanoparticles with high-encapsulation efficiency that can be injected intra-articularly and retained in the joint cavity for extended periods, releasing rapamycin at a steady rate. These RNPs were effective in preventing chondrocyte senescence and maintaining the balance between anabolic and catabolic activity, thereby aiding in preventing OA. We evaluated the efficacy of rapamycin in the NP formulation to prevent OA after articular joint injury. Intra-articular injection of RNPs significantly reduced the phenotypes of articular cartilage degeneration, synovial inflammation, osteophyte hyperplasia, and pain in a mouse DMM model. Our study highlights the potential of intra-articular injection of RNPs as a promising therapeutic strategy for clinical applications in the treatment of OA.

## Materials and methods

### Materials

Poly(_D, L_-lactic-co-glycolic) acid (PLGA, 50:50) with a molecular weight of 50 kDa was purchased from Huashi Tech. (DG-105050, Shenzhen, China). Rapamycin (99.94%) was procured from MedChemExpress (HY-10219, China). Insulin/transferrin/selenium was obtained from Gibco (41400045, CA, USA) and TGF-β1 from Novoprotein. The Calcein-AM/PI double staining kit and CCK-8 were supplied by Dojindo (Kumamoto, Japan). Trizol and PrimeScript RT reagent Kit were provided by Takara (Shiga, Japan). The Whole Cell Lysis Assay and bicinchoninic acid (BCA) Protein quantification Assay kit were purchased from Key GEN (Nanjing, China) and the RNeasy Mini kit was provided by Qiagen (Germantown, MD, USA). The SYBR qPCR Master Mix was purchased from Vazyme (Nanjing, China). Rabbit anti collagen II polyclonal antibody (IHC-P, Abcam, ab34712), rabbit anti collagen II monoclonal antibody (WB, Abcam, ab188570), rabbit anti sox9 monoclonal antibody (CST, #82630), rabbit anti MMP13 polyclonal antibody (Abcam, ab39012), rabbit anti TNF-α polyclonal antibody (Abclonal, A0277), mouse anti β-actin (MG3) monoclonal antibody (Ray antibody Tech. RM2001), Alexa Fluor 594 donkey anti-rabbit IgG (H + L) (Thermo Fischer, A21207), goat anti-rabbit IgG H&L (Horseradish peroxidase, HRP) (Abcam, ab205718), goat anti-mouse IgG(H + L)-HRP (Ray antibody Tech. RM3001) were used as primary and secondary antibodies. All solvents and products were used as received.

### Synthesis and characterization of RNPs

RNPs were synthesized using the emulsification and volatilization method. Briefly, 120 mg of PLGA was dissolved in 4 mL of dichloromethane (DCM) with or without rapamycin (3, 6, 9, 12, 15 mg). The PLGA solution was added dropwise to 20 mL of aqueous PVA solution at various concentrations (0.25%, 0.5%, 1%, 2%), followed by stirring for 30 min to form an oil-in-water (O/W) emulsion. Ultrasound (10 min, 100 W) was used to disperse the emulsion. The emulsified dispersion was then continuously stirred at 1,500 rpm at room temperature until the elution and evaporation of organic solvent were completed. Nanoparticles were collected by centrifugation at 12,000 rpm for 20 min at room temperature and washed three times with deionized water to remove excess PVA. The nanoparticles were then resuspended in deionized water and flash frozen in liquid nitrogen, followed by lyophilization. PLGA microspheres were synthesized following the same method as nanoparticles without ultrasonification. The lyophilized particles were used for in vitro and in vivo studies by suspending them in 1x phosphate-buffered saline (PBS) according to designed concentrations and sterilizing them under UV for 30 min. The size, PDI, and zeta potential of PLGA nanoparticles and PLGA microspheres were determined using a zetasizer (ZS90, Malvern, UK), after dispersing 200 µg particles in 1 mL deionized water. The morphology of PLGA particles were observed by SEM (Merlin, Zeiss, German).

### Encapsulation efficiency

To determine the encapsulation efficiency (EE) of rapamycin in PLGA particles, a standard curve was first prepared by adding different concentrations of rapamycin in methanol solution. The concentration of rapamycin was then determined with an Agilent 1260 high-performance liquid chromatography system (consisting of a G7111A Pump, G7115A DAD WR, G7129A vialsampler) equipped with a Diamonsil C18 column (150 mm by 4.6 mm, 5 μm; Agilent Technologies Inc. CA, USA). The mobile phase consisted of methanol and water (80:20, v/v) delivered at a flow rate of 1.0 mL/min. The UV detection wavelength was set to 278 nm. The column temperature was maintained at 40 °C, and the injection volume was 20 µL. The EE and LC was calculated using the following equations,

$$Encapsulation{\text{ }}Efficiency(\% )=\frac{{Amount{\text{ }}of{\text{ R}}apamycin{\text{ }}encapsulated}}{{Amount{\text{ }}of{\text{ R}}apamycin{\text{ added}}}} \times 100\%$$

$$Loading{\text{ C}}apacity(\% )=\frac{{{\text{Weight }}of{\text{ R}}apamycin{\text{ }}encapsulated}}{{Weight{\text{ }}of{\text{ R}}apamycin{\text{ }}and{\text{ }}PLGA{\text{ }}particles}} \times 100\%$$

### In vitro release profile of RNPs

The release of rapamycin from the PLGA nanoparticles was monitored in PBS (pH 7.4) containing 10% isopropanol at 37 °C under gentle shaking. In brief, 4 mL of PLGA nanoparticles (1 mg/mL) were transferred into a dialysis bag (10-kDa cutoff; Sigma-Aldrich) and immersed in 20 mL of PBS. At predetermined time intervals, 1 mL of release buffer was collected for measurement, and the same amount of fresh buffer was replenished. The concentration of released rapamycin was determined with an Agilent 1260 high-performance liquid chromatography system.

### Cell line and culture conditions

The ATDC5 mouse chondrogenic cell line was obtained from Riken BioResource Center (Tsukuba, Japan) and was cultured in DMEM/F12 medium (Gibco, USA) supplemented with 10% fetal bovine serum (FBS, Biological Industries, ISRAEL) at 37 °C under 5% CO_2_. The SW1353 human chondrosarcoma cells were purchased from ATCC and cultured in DMEM medium (Gibco, USA) supplemented with 10% FBS at 37 °C under 5% CO_2_. Primary articular chondrocytes were isolated from the articular cartilage of newborn mice (within 3 days). Rib cartilage from several mouse pups was dissected under a stereo light microscope, and the articular cartilage was incubated in 0.25% trypsin for 30 min. The cartilage was then digested in 0.1% collagenase type II (Worthington, USA) for 6 h, and chondrocytes were seeded in a T25 cell culture flask at a density of 2 × 10^5^ cells per flask and cultured at 37 °C with 5% CO_2_ in DMEM/F12 medium supplemented with 1% penicillin-streptomycin (P/S) solution (Gibco) and 10% FBS. For the chondrogenic differentiation assay, cells were cultured in differentiation medium containing DMEM, streptomycin (100 µg/mL), penicillin (100 U/mL), 1% insulin-transferrin-selenous acid (ITS) Premix (Gibco), _L_-proline (40 µg/mL), TGF-β1 (Novoprotein, 10 ng/mL), 0.1 µM dexamethasone and ascorbate 2-phosphate (50 µg/mL) in a 24-well plate. The media were refreshed every two days.

### Cell culture in 3D hydrogels

Photocrosslinkable methacrylated glycol chitosan (MeGC) was prepared following a previously established protocol [51]. Briefly, primary articular chondrocytes or ATDC5 cells at a density of 2 × 10^6^ cells/mL and rapamycin or RNPs were mixed in MeGC (2% w/v) solution and 6 µM riboflavin. The mixture was exposed to blue light (LY-A180, Carent, Hangzhou, China) for 40 s per 40 µL. Then the hydrogels were cultured in uncoated 48-well plates with 500 µL of chondrogenic medium.

### Cell viability assay

Primary articular chondrocytes were seeded at the density of 5 × 10^3^ cells/well in 96-well plates with DMEM/F12 medium (containing 10% FBS and 1% P/S), and cultured with various concentrations of free rapamycin and RNPs (0, 1, 5, 10, 20 nM rapamycin content). Live/Dead staining and CCK-8 assays were performed on day 2. Each well was washed with PBS and incubated with a Live/Dead solution containing 2 µM Calcein-AM and 4.5 µM PI for 15 min at 37 °C. The results were then observed using an Eclipse TE2000-U microscope (Nikon, Tokyo, Japan). Live cells were stained green, and dead cells were stained red. For CCK-8 assays, on day 2, cells were mildly rinsed with PBS for three times. Then, an equal volume of a mixture containing 100 µL culture medium and 10 µL CCK-8 kit was added to each well, and the plated were kept in dark. Finally, the optical density (OD) was measured at 450 nm after incubating at 37 °C for 1 h on a Synergy HTX micro-plate reader (Agilent, Santa Clara, CA, USA).

### Cellular uptake

Mouse primary chondrocytes were seeded in 35-mm confocal dishes at a density of 2 × 10^4^ cells per well and cultured overnight. Then, cells were incubated with DID - PLGA nanoparticles and DID-PLGA microspheres for 12 h in culture medium, washed with PBS, and fixed with 4% paraformaldehyde. DAPI was added to stain nuclei for 20 min. The cells were then observed under a confocal laser scanning microscope (FV3000, OLYMPUS, Tokyo, Japan). For flow cytometric analysis (fluorescence-activated cell sorting (FACS) analysis), mouse primary chondrocytes were seeded in 12-well plates at a density of 1 × 10^5^ cells per well and cultured for 24 h. DID-PLGA nanoparticles and DID-PLGA microspheres were added to the culture medium and incubated with the cells at 37 °C for 12 h. The cells were then collected and resuspended in PBS for FACS analysis with a flow cytometer (CytoFLEX, Beckman Coulter, Brea, CA, USA). The resulting data were analyzed using the FlowJo software (BD Biosciences, Franklin Lakes, NJ, USA).

### ATDC5 chondrogenic differentiation

ATDC5 were induced with chondrogenic differentiation medium for 7 days, in the absence or presence of rapamycin and RNPs. Cellular proteins and mRNAs were extracted after 7 days and 96 h, respectively. Cells were stained with Safranin O, Alcian blue and Toluidine blue to assess differentiation. Safranin O and Toluidine blue staining were performed as described previously [52]. 1% (w/v) Alcian Blue 8GX (Sigma, A5268) was dissolved in 3% (v/v) acetic acid. The medium was removed from 24-well plates and the cells were carefully washed twice with a fixed volume of PBS. Aspirate the medium and rinse with PBS three times. The cells were then fixed with 4% paraformaldehyde for 20 min, soaked in 0.1 N HCL solution for 5 min, stained with Alcian blue overnight, rinsed with 0.1 N HCL three times to remove the background unspecific staining, and then dried.

### Stressful induction for primary articular chondrocytes

Primary articular chondrocytes were induced with IL-1β (10 ng/mL) or 200 µM H_2_O_2_ for 24 h, and then treated with rapamycin and RNPs (1 µM). Cellular proteins and mRNAs were extracted after 96 h and 48 h, respectively. Alcian Blue staining and toluidine blue staining was used to assess the effects of rapamycin treatment.

### SA-β-gal staining

Primary articular chondrocytes at passage 3 were seeded into 6-well culture plates. After treatment with 200 µM H_2_O_2_ for 24 h, the cells were maintained in 50 µM H_2_O_2_ for an additional 96 h in the absence or presence of rapamycin and RNPs. SA-β-gal activity was detected using the in situ β-galactosidase staining kit (#9860, CST) following the manufacturer’s instructions. The cells were washed twice with PBS and fixed with the 4% paraformaldehyde for 15 min, followed by incubation with the SA-β-gal detection solution at 37 °C for 2 h. The cells were then washed and analyzed under a microscope (ZEISS, German) at five random sites.

### Animals

Animal experiments were approved by the Ethical Committee for Animal Research of Southern Medical University and conducted based on the state guidelines from the Ministry of Science and Technology of China. 10-week-old male C57BL/6 mice were purchased from the Laboratory Animal Center of Southern Medical University, and group-housed at 23° to 25 °C with a 12-h light/dark cycle and free access to water and standard laboratory pellets. To induce OA, 10-week-old male mice were subjected to DMM surgery or sham surgery at the right knee as previously described [53]. Briefly, DMM surgery involved opening the joint capsule immediately after anesthesia, cutting the medial meniscotibial ligament to destabilize the meniscus, and removing the anterior half of the medial meniscus appendixes associated with 1/3 of the meniscus. Sham surgery involved opening the joint capsule without any further damage. 40 male C57BL/6 mice were randomly assigned to five groups: Sham, DMM, DMM + PLGA nanoparticles (NPs), DMM + free rapamycin (Rapa), DMM + RNPs (n = 8 per group). Treatments were administered *via* intra-articular injection of 10 µL of PBS, 1% DMSO-PBS, NPs (PLGA nanoparticles equal to RNPs group), free rapamycin (10 µM rapamycin), or RNPs (10 µM rapamycin) once weekly, initiated one week after DMM surgery. Mice were euthanized at 8 weeks after surgery.

### In vivo fluorescence imaging and analysis

Rapamycin was replaced with DID perchlorate (Bioss, D-9110) during the fabrication of RNPs and RMPs to yield DID-PLGA nanoparticles and DID-PLGA microspheres, respectively. 15 male C57BL/6 mice were randomly assigned to three groups: free DID (DID), DID-labeled NPs (DID-NPs) and DID-labeled MPs (DID-MPs), n = 5. One week after DMM surgery, mice were injected with free DID, DID-PLGA nanoparticles and DID-PLGA microspheres, and subsequently anesthetized with isoflurane. The distribution of DID in vivo was analyzed at the predetermined time points using a fluorescence imaging system (Ami HT/Ami HTX, Spectral Instruments).

### Western blot assay

The cells were lysed using a whole protein extraction kit (KeyGEN BioTECH, Nanjing, China, KGP250), and the protein concentration was determined using a BCA protein assay. The protein extracts were electrophoresed in 10% SDS-PAGE and transferred onto a nitrocellulose filter (NC) membrane, which was blocked by 5% skim milk in Tris-buffered saline with tween 20 (TBST) at room temperature for 1 h. Then the membranes were immunoblotted with primary antibodies. For detection, HRP-conjugated secondary antibodies and a chemiluminescent HRP substrate kit (EpiZyme, Shanghai, China, SQ2O2) were used. In order to detect multiple targets, the western blot fast stripping buffer (EpiZyme, Shanghai, China, PS107) was used due to the similar molecular weight of the target bands.

### Evaluation of OA-related pain

To evaluate knee joint pain in mice following DMM surgery, von Frey filaments were employed [8]. Each mouse was placed on a wire-mesh platform under a 4 × 3 × 7 cm^3^ cage to restrict its movement. The mice were trained to adjust to this condition every day starting from 7 days before the test. During the test, a set of von Frey fibers (KEW BASIS, KW-CT, China) were applied to the plantar surface of the hind paw. The threshold force required to elicit withdrawal of the paw (median 50% withdrawal) was determined five times on each hind paw with sequential measurements separated by at least 30 min.

### micro-CT imaging and analysis

Knee samples from mice were fixed in 4% paraformaldehyde and imaged using micro-CT (Scanco Medical, µCT 40) with a voltage of 80 kV and a resolution of 15 μm per pixel. The reconstructed images were analyzed for periarticular osteophytes volume. The software Mimics 21.0 was used for three-dimensional knee reconstruction and image capture. The region of interest (ROI) was selected from periarticular osteophytes and marked in red. ROI size was calculated blinded on all four condyles of the knees (medial and lateral side of the tibia and femur), and the average was used for statistical analysis.

### Histomorphometric analysis

At week 8 post-surgery, knee samples were fixed in 4% paraformaldehyde solution 48 h and decalcified in 0.5 M EDTA, pH 7.4, on a shaker for 2 weeks. Paraffin sections were prepared for Safranin O/Fast green or Hematoxylin and Eosin (H&E) staining. The severity of cartilage degeneration and synovitis was evaluated using the OARSI scoring system [53], Mankin scoring system [8] and Krenn scoring system [54].

### Immunohistochemical (IHC) and immunofluorescence (IF) staining

The primary antibodies were applied and incubated overnight at 4 °C, followed by the application of HRP-conjugated secondary antibodies, which were incubated for 1 h at room temperature. For IHC staining, the brown color was developed with DAB kit (ZL1-9018, Origene) and counterstained with Hematoxylin. For IF staining, DAPI (D9542, Sigma Aldrich) was used to stain the nuclei.

### Quantitative reverse transcription polymerase chain reaction qRT-PCR assay

Total RNA was extracted from cells using Trizol reagent (Takara, Shiga, Japan, 9109) and the RNeasy Mini kit (Qiagen, Germantown, MD, USA, 74,104). The cDNA was reversely transcribed from the extracted RNA with the PrimeScript RT reagent Kit (TAKARA, Japan). The relative gene levels were quantified using a qPCR machine (Applied Biosystems Step One Plus, ThermoFisher, Waltham, MA, USA) with the presence of 20 µL SYBR Green for 45 cycles, and normalized to the expression levels of a housekeeping gene (β-actin). The detailed primer sequences are listed in Table S1.

### Statistical analysis

Data are expressed as means ± SD and analyzed by one-way analysis of variance (ANOVA) with Dunnett’s or Tukey’s *post hoc* test, and two-way ANOVA with Bonferroni’s or Tukey’s *post hoc* test for multiple comparisons using Prism 8 software (GraphPad Software). For cell culture experiments, observations were repeated independently at least three times. Values of *P* < 0.05 were considered statistically significant.

### Electronic supplementary material

Below is the link to the electronic supplementary material.

**Additional file 1:** Table S1. List of primer sequences. Figure S1. Preparation and characterization of RNPs and RMPs. Figure S2. Stability of RNPs and RMPs suspended in physiological saline. Figure S3. In vitro cellular uptake. Cellular uptake of RNPs and RMPs by flow cytometry in RAW 264.7 cells (n = 3). Figure S4. Quantification of RNPs and RMPs visualized by CLSM in mouse primary articular chondrocytes (n = 4). Figure S5. Toluidine blue staining of ATDC5 cells cultured in chondrogenic medium for 7 days treated with 0, 5, 10, 20 and 30 nM rapamycin, respectively. Figure S6. The expression of proteins (Col II, p-S6, MMP13 and TNF-α) in SW1353, which were induced with IL-1β for 24 h. Figure S7. Quantification of Toluidine blue and Alcian blue staining (n = 3). Figure S8. Distribution of fluorescence signals after 24 h of injecting PLGA nanoparticles. Figure S9. Hematoxylin and eosin staining of heart, liver, spleen, lung and kidney harvested from mice at week 8 post surgery.

## Data Availability

The data supporting the findings of this study are available from the corresponding author upon reasonable request.
