# Supplementary material for: Exploring the translational potential of PLGA nanoparticles for intra-articular rapamycin delivery in osteoarthritis therapy
Source: J Nanobiotechnology. 2023 Oct 4;21:361. doi: 10.1186/s12951-023-02118-4 (PMC10548624; doi:10.1186/s12951-023-02118-4)
Supplement: Supplementary file 1 — Additional file 1: Table S1. List of primer sequences. Figure S1. Preparation and characterization of RNPs and RMPs. Figure S2. Stability of RNPs and RMPs suspended in physiological saline. Figure S3. In vitro cellular uptake. Cellular uptake of RNPs and RMPs by flow cytometry in RAW 264.7 cells (n = 3). Figure S4. Quantification of RNPs and RMPs visualized by CLSM in mouse primary articular chondrocytes (n = 4). Figure S5. Toluidine blue staining of ATDC5 cells cultured in chondrogenic medium for 7 days treated with 0, 5, 10, 20 and 30 nM rapamycin, respectively. Figure S6. The expression of proteins (Col II, p-S6, MMP13 and TNF-α) in SW1353, which were induced with IL-1β for 24 h. Figure S7. Quantification of Toluidine blue and Alcian blue staining (n = 3). Figure S8. Distribution of fluorescence signals after 24 h of injecting PLGA nanoparticles. Figure S9. Hematoxylin and eosin staining of heart, liver, spleen, lung and kidney harvested from mice at week 8 post surgery. [file 12951_2023_2118_MOESM1_ESM.pdf]

Exploring the Translational Potential of PLGA Nanoparticles for Intra-articular Rapamycin Delivery in Osteoarthritis Therapy

Jian-Chao Ma[1], Tingting Luo[1], Binyang Feng[1], Zicheng Huang[1], Yiqing Zhang[1], Hanqing Huang[1], Xiao Yang[1], Jing Wen[1], Xiaochun Bai[1,*], Zhong-Kai Cui[1,*]

[1] Department of Cell Biology, School of Basic Medical Sciences, Southern Medical University, Guangzhou, 510515, China

*Correspondence:

Xiaochun Bai, baixc15@smu.edu.cn

Zhong-Kai Cui, zhongkaicui@smu.edu.cn

Table S1. List of primer sequences.

Gene	Forward primer (5'to3')	Reverse primer (5'to3')
Col2a1	CTGGTGGAGCAGCAAGAGCAA	CAGTGGACAGTAGACGGAGGAAAG
Sox9	AGGAGAGCGAGGAAGATAAG	ACGTGTGGCTTGTTCTTG
Mmp13	CAGTTGACAGGCTCCGAGAA	CGTGTGCCAGAAGACCAGAA
β-actin	GGGACCTGACTGACTACCTC	TCATACTCCTGCTTGCTGAT

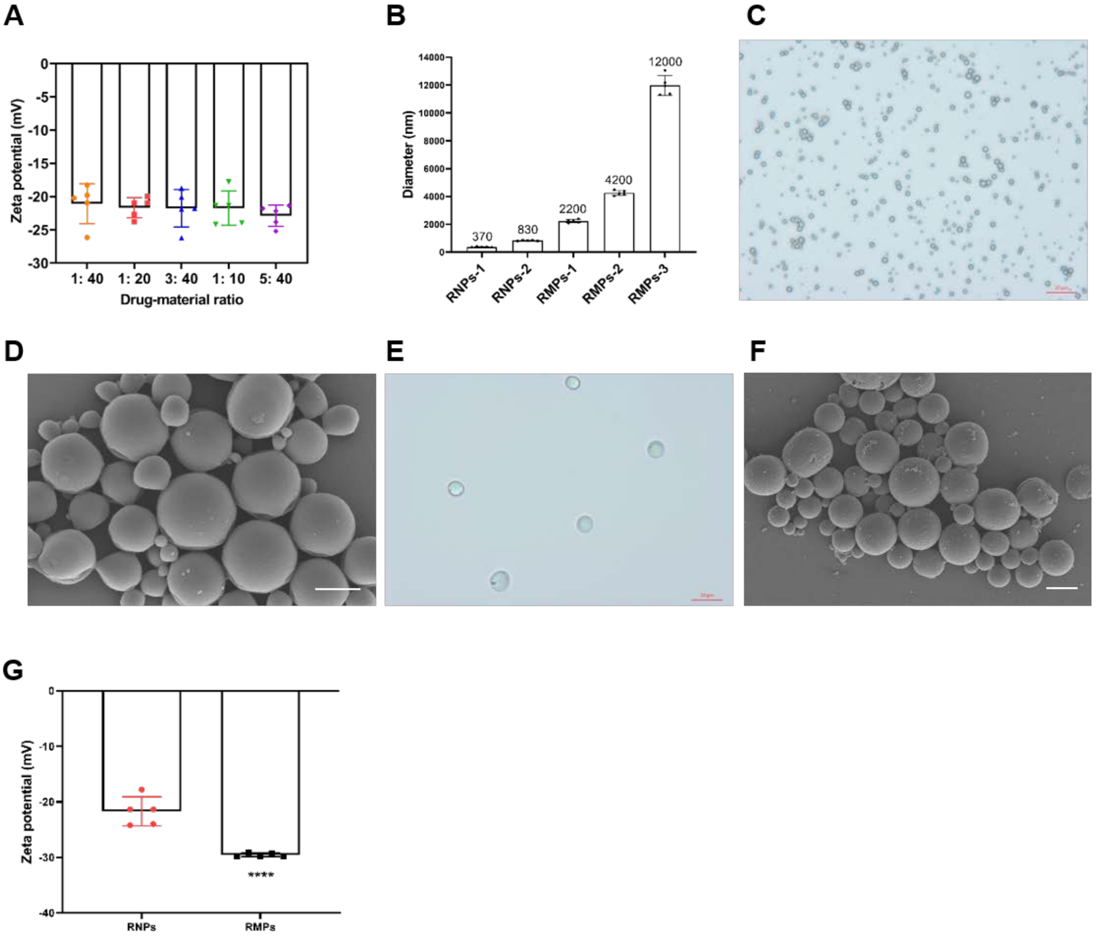

Figure S1. Preparation and characterization of RNPs and RMPs. (A) Zeta potential of RNPs with different drug-to-material ratios. (B) Average diameter of RNPs and RMPs (0.5% PVA concentration, drug-to-material ratio of 1:10). Average diameter of RNPs-1, RNPs-2, RMPs-1, RMPs-2 and RMPs-3 measured by dynamic light scattering. Average diameter of RMPs-4 obtained from Scanning Electron Micrograph measured by Image J (n = 3). (C) Upright light microscope image of RMPs-1, Scale Bar 20 μm. (D) Scanning Electron Micrograph of RMPs-1, Scale Bar 2 μm. (E) Upright light microscope image of RMPs-3, Scale Bar 20 μm. (F) Scanning Electron Micrograph of RMPs-3, Scale Bar 10 μm. (G) Zeta potential of RNPs and RMPs-1 (0.5% PVA concentration, drug-to-material ratio of 1:10).

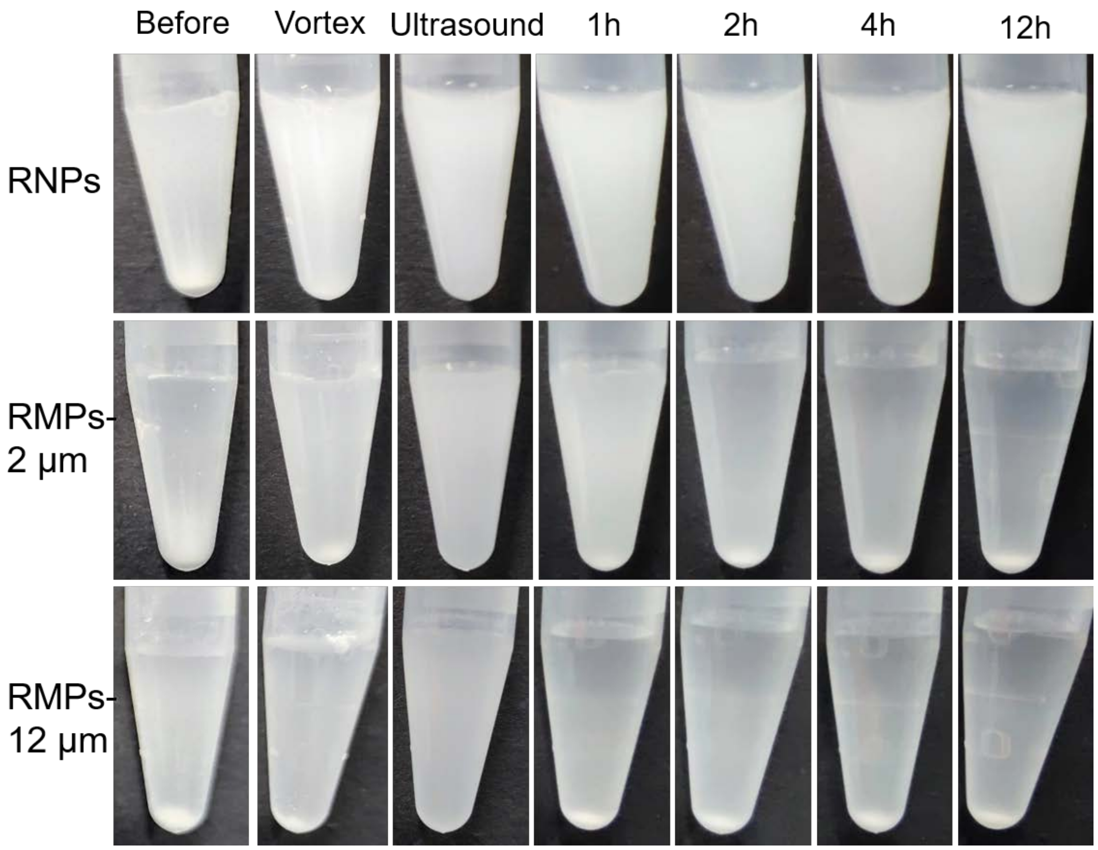

Figure S2. Stability of RNPs and RMPs suspended in physiological saline. Photographs in sequence showing the suspensions before vortex or ultrasound, after vortex, after ultrasound, 1, 2, 4, and 12 h after ultrasound, respectively.

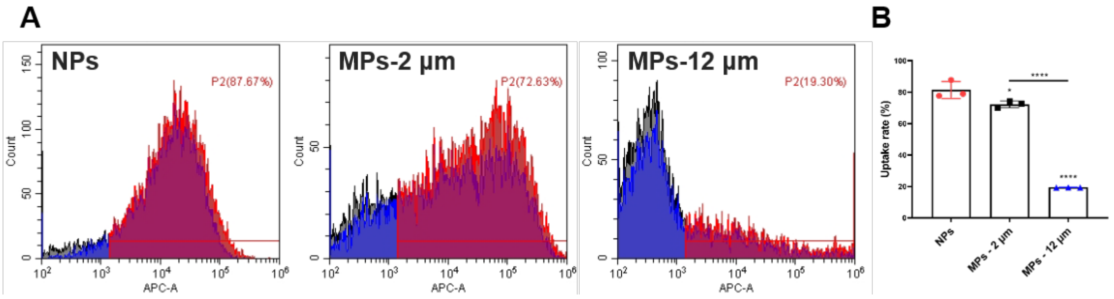

Figure S3. *In vitro* cellular uptake. Cellular uptake of RNPs and RMPs by flow cytometry in RAW 264.7 cells (n = 3). Statistical analysis was performed using one-way ANOVA with Tukey's *post hoc* analysis. Data are presented as means ± SD. **P* < 0.05, ***P* < 0.01, ****P* < 0.001, *****P* < 0.0001.

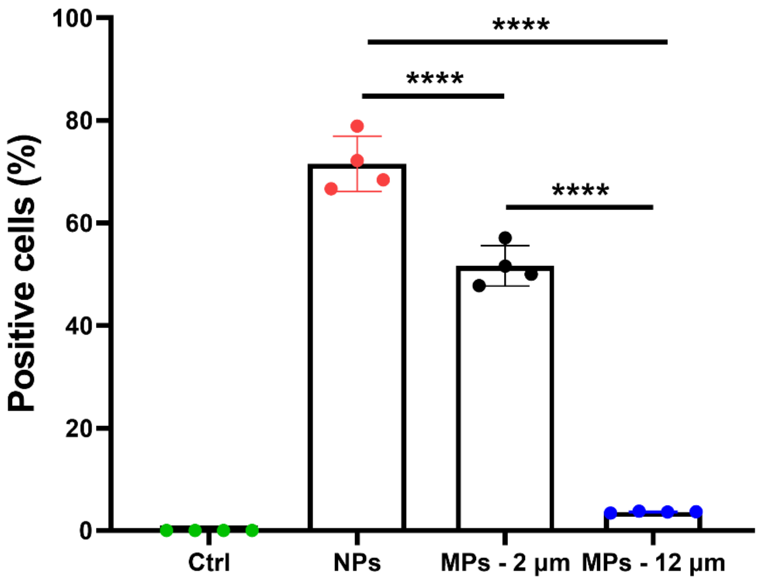

Figure S4. Quantification of RNPs and RMPs visualized by CLSM in mouse primary articular chondrocytes, data are presented as means ± SD. ****P < 0.0001, (n = 4).

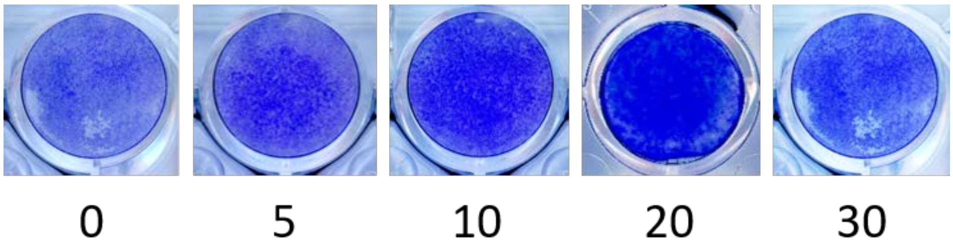

Figure S5. Toluidine blue staining of ATDC5 cells cultured in chondrogenic medium for 7 days treated with 0, 5, 10, 20 and 30 nM rapamycin, respectively.

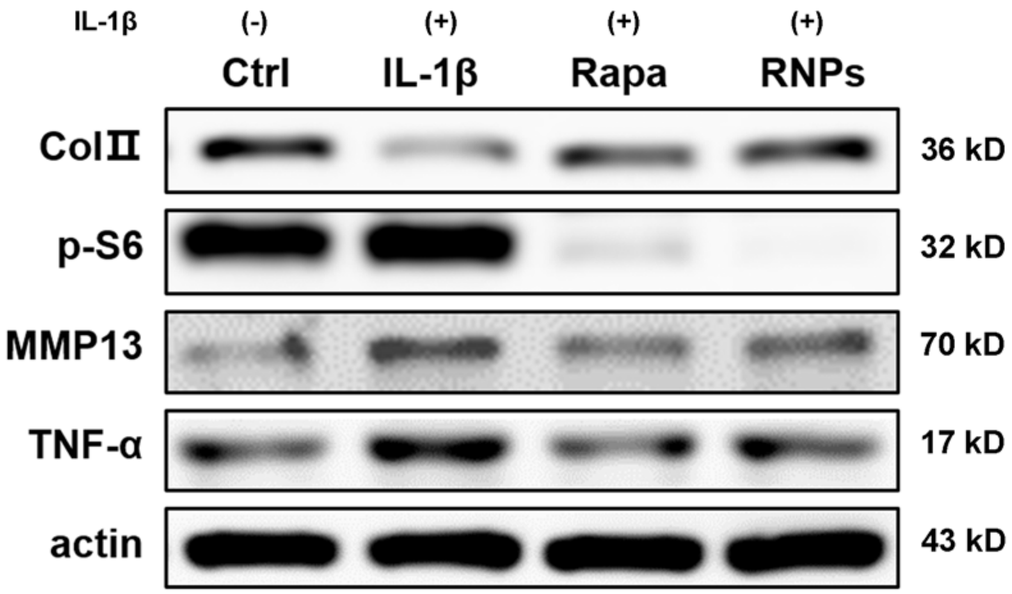

Figure S6. The expression of proteins (Col II, p-S6, MMP13 and TNF-α) in SW1353, which were induced with IL-1β for 24 h.

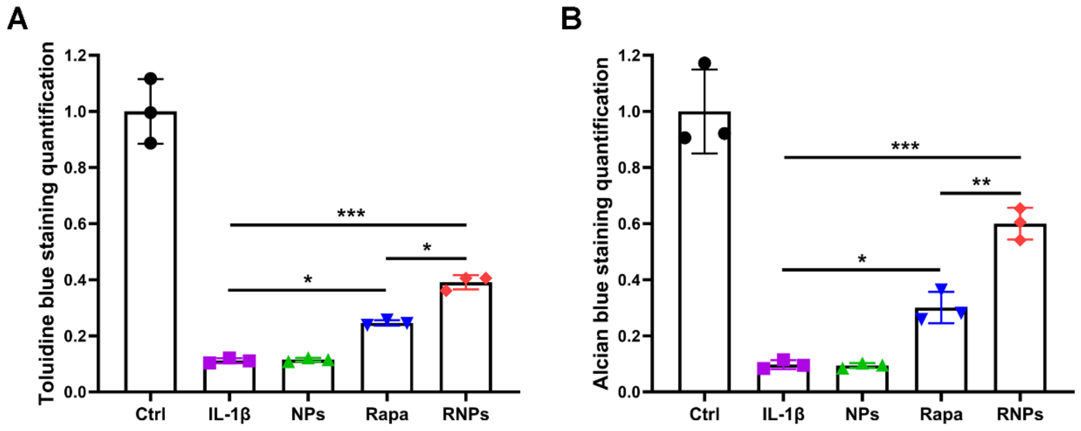

Figure S7. Quantification of Toluidine blue and Alcian blue staining, data are presented as means ± SD. **P* < 0.05, ***P* < 0.01, ****P* < 0.001, (n = 3).

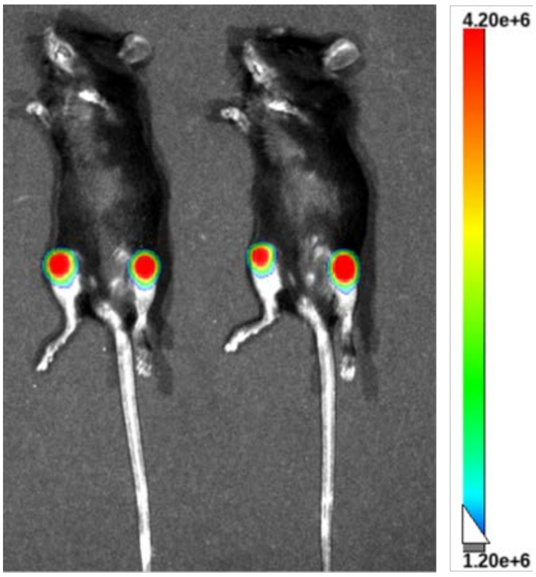

Figure S8. Distribution of fluorescence signals after 24 h of injecting PLGA nanoparticles.

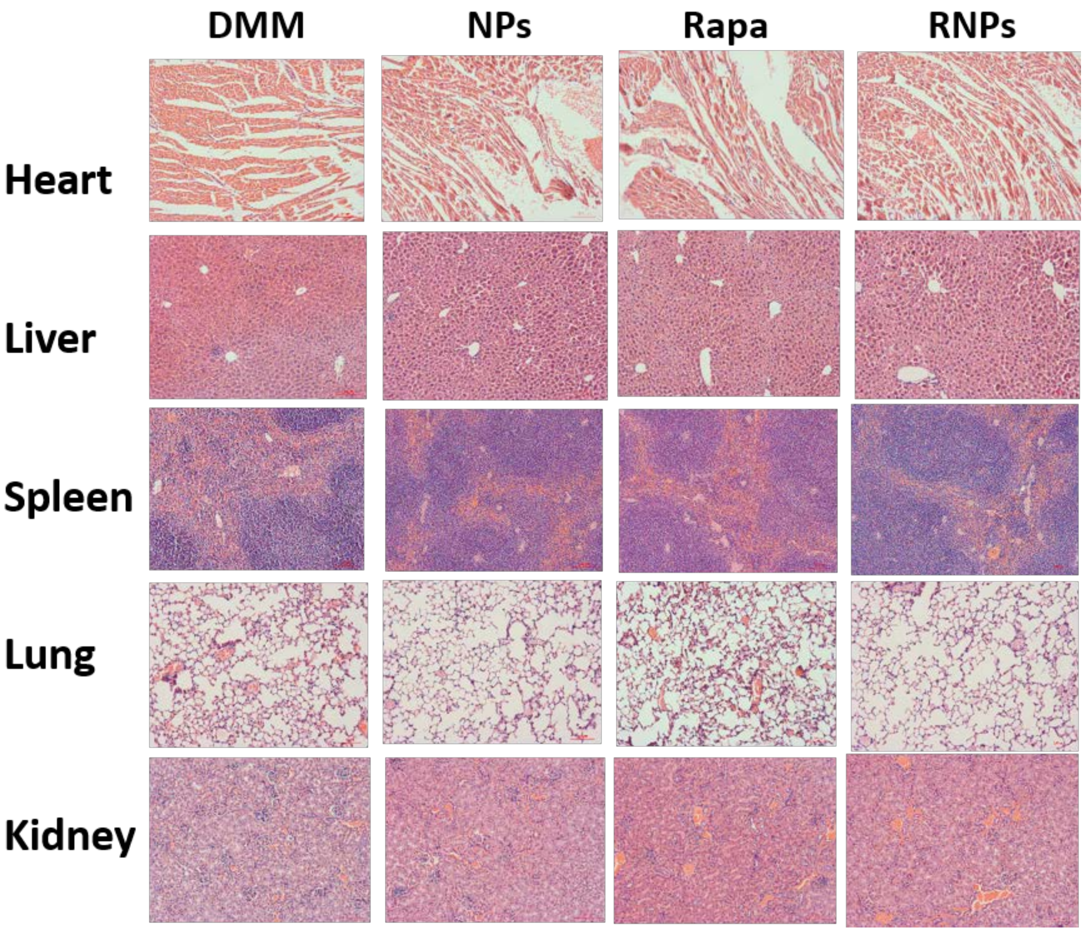

Figure S9. Hematoxylin and eosin staining of heart, liver, spleen, lung and kidney harvested from mice at week 8 post surgery.
